# Shaping the future from the small scale: dry powder inhalation of CRISPR-Cas9 lipid nanoparticles for the treatment of lung diseases

**DOI:** 10.1080/17425247.2023.2185220

**Published:** 2023-03-12

**Authors:** Simone P. Carneiro, Antonietta Greco, Enrica Chiesa, Ida Genta, Olivia M. Merkel

**Affiliations:** a Department of Pharmacy, Pharmaceutical Technology and Biopharmaceutics, Ludwig-Maximilians-University of Munich, Butenandtstraße 5, 81377 Munich, Germany; b University School for Advanced Studies (IUSS), Piazza della Vittoria 15, 27100 Pavia, Italy; c Department of Drug Sciences, University of Pavia, V.le Taramelli 12, Pavia, Italy

**Keywords:** gene therapy, CRISPR-Cas9, lipid nanoparticles, pulmonary administration, spray drying

## Abstract

**Introduction:**

Most lung diseases are serious conditions resulting from genetic and environmental causes associated with high mortality and severe symptoms. Currently, treatments available have a palliative effect and many targets are still considered undruggable. Gene therapy stands as an attractive approach to offering innovative therapeutic solutions. CRISPR-Cas9 has established a remarkable potential for genome editing with high selectivity to targeted mutations. To ensure high efficacy with minimum systemic exposure, the delivery and administration route are key components that must be investigated.

**Areas covered:**

This review is focused on the delivery of CRISPR-Cas9 to the lungs, taking advantage of lipid nanoparticles (LNPs), the most clinically advanced nucleic acid carriers. We also aim to highlight the benefits of pulmonary administration as a local delivery route and the use of spray drying to prepare stable nucleic acid-based dry powder formulations able to overcome multiple lung barriers.

**Expert opinion:**

Exploring the pulmonary administration to deliver CRISPR-Cas9 loaded in LNPs as a dry powder increases the chances to achieve high efficacy and reduced adverse effects. CRISPR-Cas9 loaded in LNP-embedded microparticles has not yet been reported in the literature but has the potential to reach and accumulate in target cells in the lung, thus, enhancing overall efficacy and safety.

## 1 Introduction

Chronic respiratory diseases are a major concern in public health worldwide due to persistent conditions and high incidence and mortality rates. Chronic obstructive pulmonary disease (COPD) is one of the most serious lung diseases and accounted for more than 3.2 million deaths in 2019. Worryingly, additional data from the World Health Organization discloses that COPD is the third global cause of death. Lung cancer numbers are even more severe, and statistics from 2020 ranked the disease as the main cause of cancer death, counting 1.80 million deaths and 2.21 million new cases [1,2]. Asthma, another common lung disease especially among children, affected more than 262 million people in 2019 and resulted in 455,000 deaths [3].

Generally, inflammation in the airways is a shared condition among numerous lung disorders, which varies in regard to severity depending on the disease. Cystic fibrosis (CF), for example, is a monogenetic disease caused by mutations in the CF transmembrane conductance regulator (CFTR) gene that, consequently, affects the expression of functional CFTR protein, which plays an essential role in homeostasis and regulator processes [4]. A malfunctioning or absent CFTR protein causes patients a highly viscous, adherent and thicky mucus, which creates a severe scenario of inflammation and recurring infections that drastically affect the patients‘ quality of life or even culminate in serious consequences, such as severe bronchiectasis and, eventually, respiratory failure [5]. Asthma is another chronic respiratory disease presenting an upregulation of genes that trigger the release of proinflammatory cytokines [6]. Due to a pronounced narrowing of the small airways, symptoms such as shortness of breath, mucus hypersecretion, and broncho obstruction with airway hyperactivity and remodeling are frequently reported [2,7]. In COPD, tumor necrosis factor-alpha (TNF-α) levels are upregulated, which ultimately causes abnormal inflammatory responses to noxious particles and swelling of the airway lining, among other strong reactions [8]. Also this condition is associated with airway narrowing. The airflow limitation is usually progressive, which contributes to the emergence of persistent symptoms, such as increasing breathlessness, productive cough with phlegm, and frequent chest infections [1,9]. If not controlled, symptoms may progress to permanently damage the lungs [10]. Worryingly, long-term COPD smoking patients have an up to 4.5-fold increased risk of developing lung cancer [11].

A group of severe respiratory chronic diseases presents mutations, genetic heritage, environmental factors, or occupational exposure as a central component. COPD, lung cancer, and interstitial pulmonary fibrosis are examples of diseases that have at least one of these causes but more frequently, a combination of several factors [7,9,12].

Despite the constantly increasing burden of respiratory disorders, the treatment options are limited and mostly related to palliative therapy with several adverse effects that, consequently, result in low patient compliance. Although the currently available treatments for chronic respiratory diseases are capable of achieving considerable symptomatic improvements, a therapy designed to target a specific key component responsible for triggering the disease may represent a clinical advance.

Since all the above-mentioned disorders have an established genetic target, gene therapy has remarkable potential to offer an effective treatment. Notably, gene editing has emerged a few years ago as an outstanding platform able to correct specific mutations, as performed by Clustered regularly interspaced short palindromic repeats (CRISPR-Cas9) which was awarded the Chemistry Nobel Prize in 2020 [13]. Once the genetic target involved in the pathogenesis of specific lung diseases is known, the CRISPR-Cas9 gene editing tool is in principle capable of efficiently and selectively correcting mutations through deletions and/or repair insertion in the DNA.

For safe and effective clinical use, genome editing using CRISPR-Cas9 requires accurate and targeted delivery systems (DSs) for targeting the desired cells. Therefore, ensuring local treatment by taking advantage of a potent cargo increases the chances for successful therapy with lower adverse effects. The present work aims to review the state of the art of CRISPR-Cas9 as an attractive alternative to treat lung diseases and lipid nanoparticles (LNP) as potent and effective nanocarriers. Furthermore, we also aim to highlight the benefits of pulmonary administration of nucleic acid-based cargos using nano-DSs to effectively and locally overcome lung barriers. Ultimately, to support pulmonary administration, we review spray drying as a promising approach to preparing dry powder composed of nanoparticles embedded in microparticles with appropriate aerodynamic properties to be administered and to penetrate within the lungs.

## 2 CRISPR-Cas9

### 2.1 CRISPR-Cas9 mechanism

**Figure 1:**
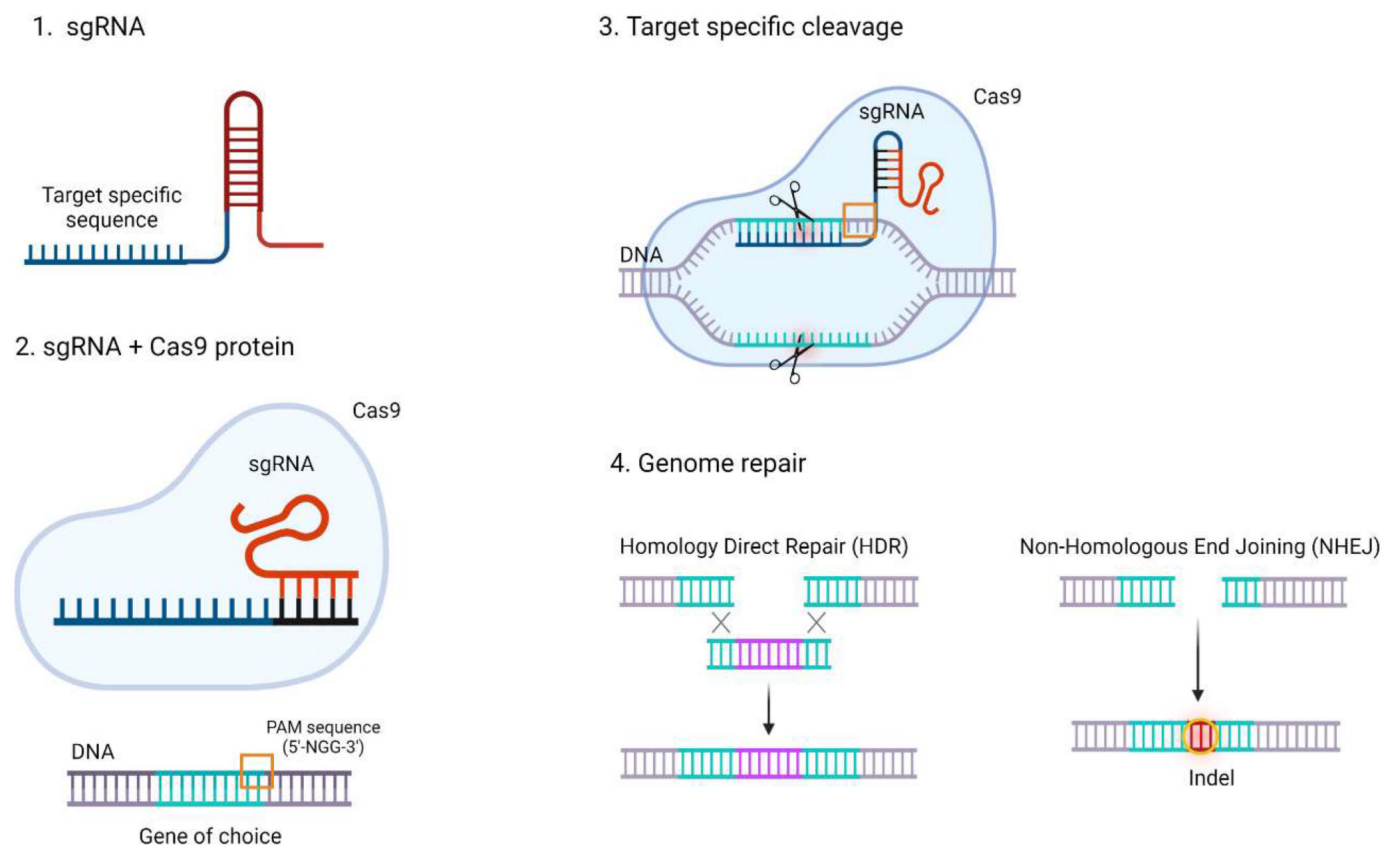
Schematic structure and mechanism of action of CRISPR-Cas9. 1. sgRNA representation. 2. sgRNA + Cas9 protein illustration: the sgRNA-Cas9 exploits the PI domain to recognize and match with PAM sequences in the DNA, (3) triggering the strand separation of the target DNA duplex and promoting sgRNA-DNA hybrid formation, which enhances the DNA DSB. 4. Consequently, the cell pathways for genome repair NHEJ and HDR are enabled and exploited for gene deletions or insertions.

CRISPR-Cas9 is composed of the protein Cas9 and a chimeric single-stranded RNA called single guide RNA (sgRNA) identified as a ribonucleoprotein (RNP) (Figure 1). Cas9 endonuclease shows a bilobed architecture composed of the Recognition (REC) lobe, including the REC and Bridge Helix (BH) domains, and the Nuclease (NUC) lobe, organized in three specific domains as protospacer adjacent motif (PAM) interacting (PI), HNH (the abbreviation derives from the characteristic histidine (H) and asparagine (N) residues of the endonuclease) and RuvC (named for an E. Coli protein involved in DNA repair) [14–18].

The activation of Cas9 occurs when the REC domain interacts with the sgRNA. The Cas9–sgRNA exploits the PI domain to recognize and match with the DNA PAM triggering the strand separation of the target DNA duplex and promoting sgRNA-DNA hybrid formation. Subsequently, after BH activation, HNH, and RuvC domains cleave the DNA forming a double-strand break (DSB) (Figure 1) [19,20]. Consequently, the cell pathways for genome repair, namely Non-Homologous End Joining (NHEJ) and Homology Direct Repair (HDR), is enabled [18,21]. NHEJ is the most frequent DNA reparation route which directly generates variable insertions or deletions at the DSB, while HDR uses homologous donor DNA sequences from chromatids, chromosomes, or exogenous DNA molecules to produce tailored deletions, insertions, or substitutions of a base at a DSB site or between two DSBs. Based on the gene editing aims, specific modifications are preferred for replacing the broken DNA with a precise sequence, while the random repair of the cleaved DNA is chosen for the generation of gene knockouts [21–23]. These pathways are governed by the cell cycle thus having the ability to manage the DNA repair mechanisms, preferring either NHEJ or HDR. Specifically, NHEJ rules the DNA repair during the cell cycle phases G1, G2, and S, while HDR happens exclusively in the late S phase and G2 phase when the DNA is entirely replicated, and sister chromatids can behave as repair templates. Therefore, cell cycle synchronization is a worthwhile strategy to make the most of CRISPR-Cas9 systems ensuring their success in genome editing [21,24–26]. In these regards, the discovery of homology-independent targeted integration (HITI) has led to the generation of gene knock-in also exploiting the NHEJ pathway [27]. This new approach is more attractive concerning HDR since NHEJ is always active in the cell cycles but, at the same time, it brings many off-target modifications and the gene knock-in efficiency is less than 5%. Following recent breakthroughs such as Microhomology-mediated end joining, or the introduction of point mutations into either of the Cas9 nuclease domains which allows single-stranded nicks instead of double-stranded breaks, CRISPR-Cas is becoming an increasingly precise and smart genome editing tool [28].

### 2.2 CRISPR-Cas: advantages and limitations

CRISPR-Cas9 has revolutionized the gene editing world and is an arising therapy concept. It is an extremely simple, versatile, efficient, and precise genome editing tool since nucleic acid cleavage only occurs if a sufficient homology with the target DNA is verified [23,29]. Interestingly, CRISPR-Cas9 is unaffected by the chromatin methylation state which further streamlines DNA editing [30]. Other advantages of CRISPR-Cas9 include the possibility of multiple independent editing sites, chromosomal target modification with low toxicity, and in theory the ability to directly modify the genome in embryos notwithstanding all ethical concerns this aspect implies [31].

Despite the advantages and the abovementioned great promises, there are still some hurdles to overcome before safe application of CRISPR–Cas9 in therapy may become reality.

One of the main determinants of the safe use of the CRISPR-Cas system is the “off-target mutation” referring to unsolicited and uncontrolled changes resulting in genome instability, loss of efficacy, and activation of pathological pathways; off-target genome modifications detected in human and mammalian systems cannot be overlooked. One of the strategies adopted to address this condition was studied by Slaymaker [32] where the Cas protein was skillfully modified to enhance the ability of the Cas domains to specifically recognize and interact exclusively with the targeted DNA. Another approach is represented by the combination of laboratory techniques and Machine learning [33]. Finally, CRISPR-Cas delivery strategies are widely studied to limit the drawback of off-target effects [34].

Another drawback of CRISPR-mediated gene editing is mosaicism. Mosaicism is mainly described in embryos during the development of knockout and transgenic animal models. In this context, following the introduction of CRISPR-Cas9 into fertilized zygotes, mosaic animals have been obtained. The primary consequence is the generation of false positive models which will not be able to transmit the genetic modification to their offspring. Mosaicism detection and prevention are challenging, expensive, and extremely time-consuming [32,35,36]. However, it bears the danger of data misinterpretation and therefore needs to be addressed properly.

Being a rather novel tool, CRISPR-Cas systems lack guidelines for its application, thus generating ethical problems. The first official application of CRISPR-Cas9 to modify the genome of human embryos was for the treatment of the β-thalassemia disorder. Even though the experiment was carried out on nonviable embryos, it moved the entire world and raised serious concerns. The event caused a stir in the scientific community which convened at the Second International Summit on Human Genome Editing to discuss the ethical use of CRISPR-Cas and to firmly outlaw the generation of modified humans. However, in 2018 successful results of modifying embryos by obtaining the first human species resistant to HIV infection were announced. Besides fines and legal consequences for the scientist, this event sparked new ethical discussions about CRISPR-Cas9 applications [35,37].

### 2.3 CRISPR-Cas9 Delivery

Although CRISPR-Cas9 has a very simple structure, its delivery is challenging. The appropriate delivery strategy for CRISPR-Cas9 is crucial to guarantee effective and accurate gene editing. Indeed, to perform its editing function, the CRISPR-Cas9 system needs to overcome different biological barriers. Moreover, CRISPR-Cas9 requires the codelivery of Cas9 protein and the sgRNA, which can be achieved in three different ways [28,38]:

I) **Cas9 protein and sgRNA**: The most straightforward approach of CRISPR-Cas9 delivery consists of the RNP complex derived from the *in vitro* incubation of Cas9 protein and sgRNA. This CRISPR-Cas9 form is ready for genome editing and, the short half-life of the Cas9 protein allows the reduction of off-target modifications. However, the synthesis and purification of Cas9 are expensive processes prone to contamination with bacterial endotoxins. The Cas9 molecular weight of approximately 160 kDa is not advantageous for cellular internalization, thereby limiting its gene editing function and extracellularly eliciting an immune response by anti-Cas9 T-cells [39–41].
II) **Plasmid DNA encoding for Cas9 protein and sgRNA**: The cheapest way to deliver CRISPR-Cas9 is delivery of a single plasmid that encodes both entities. Cas9, sgRNA, and possibly, homologous recombination repair templates can be smoothly encoded by the same plasmid, resulting in large vectors (>7kb), however, which may limit cell transfection. Furthermore, the plasmid must enter the cell nucleus for transcription, the corresponding mRNA needs to be exported to reach the cytoplasm for translation of the Cas9 protein, and then the protein has to assemble with the sgRNA to form the RNP ready for genome editing. This prolonged time between cellular administration of the CRISPR-Cas9 plasmid and the start of genome editing increases the possibility of immune response and off-target modifications [42–45].
III) **Cas9 mRNA and sgRNA**: Both RNAs are easily produced *in vitro* by solid phase synthesis of the short RNA and *in vitro* transcription (IVT) of the mRNA. They are delivered to the host cell cytoplasm to be translated and assembled into the RNP CRISPR-Cas9. The major drawbacks of this approach are potential off-target genome edits and mRNA instability, partially solved by chemical modifications and addressed through adequate delivery [46,47].

CRISPR-Cas9 can be delivered to target sites by leveraging different technologies that can overcome the disadvantages discussed above. CRISPR-Cas9 delivery technologies follow the general terminology of nucleic acid delivery technologies, which are broadly classified into physical delivery, viral vector delivery, and non-viral vector delivery.

The most applied technologies to physically deliver CRISPR-Cas9 are microinjection, electroporation, and hydrodynamic tail-vain injection. In general, physical approaches for CRISPR-Cas9 delivery are difficult to apply *in vivo,* require highly specialized operators while resulting in poor throughput and compromised cell and organ integrity and functionality. Therefore, the CRISPR-Cas9 delivery through nanosized carriers is preferred.

#### 2.3.2 Viral vector delivery

Viral vector systems are the most ancient vehicles used for gene therapy [48]. Lentiviral vectors can hold big payloads as shown by cloning both genes coding for Cas9 and sgRNA into a single vector. A lentiviral vehicle’s downside is the random editing of the host cell genome leading to the probability of oncogene activation, thus limiting its application *in vivo* and in clinical trials [23,48].

Adenoviruses were employed to deliver CRISPR-Cas9 for PTEN gene editing. However, the major challenge of using adenoviruses is the activation of immune responses with consequent tissue inflammation and viral vector inactivation [49,50].

Adeno-associated viruses (AAVs) are stable vehicles able to modify the target genome with low toxicity and fewer off-target editing. AAVs can efficiently deliver the CRISPR-Cas9 system and can thus also be used as a donor model for gene knock-in. However, the cloning capacity is very low and the prolonged expression promotes off-target mutations [51,52]. Moreover, being a virus derivate, AAV vectors can readily activate the immune system resulting in immunogenic responses or neutralization; nonetheless, AAVs have been described for successful *in vivo* genome editing in the lung using a reporter gene [53].

Although viral vectors are not directly associated with any damage to patients, the potential for triggering infections and pathological conditions is the main concern that leads to a preference for the development of non-viral drug DSs. Furthermore, once within the body, viral vectors are not able to discriminate against target cells and only few viruses have a defined tropism. Besides, the prolonged expression of CRISPR-Cas9 components delivered by viral vectors is a huge stumbling block to overcome. Therefore, the best alternative to deliver CRISPR-Cas9 accurately, safely, and efficiently is achieved with non-viral vectors [54–56].

#### 2.3.3 Non-viral vector delivery

Non-viral vectors are the results of multidisciplinary investigations aimed at producing innovative materials and vectors capable of correctly delivering nucleic acids in general and CRISPR-Cas9 specifically to improve its genome editing activity. Based on materials and characteristics, non-viral vectors are versatile systems, easy to modify, and capable of addressing pharmacokinetics limitations based on payload characteristics and route of administration [48]. In recent years, nano-sized carriers have attracted the attention of numerous research groups and pharmaceutical companies as drug DSs, including the delivery of different forms of CRISPR-Cas9 systems.

Polymers include a wide pool of materials from different origins broadly used to produce nano-carriers and sometimes even actively contributing to the therapeutic effect. Cationic polymers such as chitosan and polyethyleneimine (PEI) are extensively studied for nucleic acid delivery due to their versatile structure and high loading capability [54,57,58]. Indeed, cationic polymers can electrostatically interact with the negatively charged DNA or mRNA encoding for CRISPR-Cas9 to obtain nanosized formulations able to protect the payload and ensure its selective delivery. By regulating the electrostatic interaction between polymer and DNA or RNA it is possible to properly manage the nanocarrier features to efficiently achieve the target. The main advantages of polymeric nanoparticles for CRISPR-Cas9 delivery are the maximization of the editing activity, low immunogenicity, easy production and storage stability of formulations. In the past years, various polymeric formulations for CRISPR-Cas9 delivery have been used in the scientific community [59–63].

But also nanostructured DNA can be used as a carrier for CRISPR-Cas9 delivery. DNA nano-clews are nanosized structures consisting of degradable deoxyribonuclease rings and acid-responsive deoxyribonuclease I nano-capsules able to deliver the CRISPR-Cas9 system. These DNA nanostructures were obtained with an innovative approach which consists in synthesizing DNA nanowires by rolling circle amplification with palindromic sequences able to self-assemble into nanocarriers [64]. The first formulation of CRISPR-Cas9 in DNA nano-clews structures dates back to 2015 when Sun and collaborators [64] efficiently loaded and delivered the Cas9 protein and sgRNA to the nucleus of human cells. Cellular uptake and endosomal escape of these DNA-based nanostructures were enhanced by coating the carriers with the cationic polymer PEI resulting in a genome editing efficiency of approximately 28%. Despite general improvements in CRISPR-Cas9 delivery over the years, further investigations regarding cytotoxicity and immunogenicity are required [28].

Another interesting aspect related to CRISPR-Cas9 delivery includes gold nanoparticles as non-viral carriers. The nanoparticle surface is modified with functional groups such as the sulfhydryl (-SH) group able to interact with CRISPR-Cas9 RNP or plasmid DNA. Many gold-based CRISPR-Cas9 formulations are currently studied both *in vitro* and *in vivo* for the treatment of different diseases such as illnesses related to X fragile syndrome [65–67].

The classification of non-viral vectors includes also naturally occurring vesicles, named exosomes, made of cellular membrane phospholipids and able to load different therapeutics, including nucleic acids and proteins, resulting in biocompatible candidates for CRISPR-Cas9 delivery. This delivery strategy allows to load CRISPR-Cas9 in different forms (see 2.3). Through tailored technologies, it is possible to modify the exosome surface to improve selective interactions with target cells and organs. The major detriments connected with exosomes are the low cargo encapsulation efficiency and the elaborate and costly preparation process. Interestingly, exosomes can be derived from different cells to increase the preferential uptake within specific targets [38]. The work of Usman and colleagues [68] describes exosomes derived from red blood cells to deliver CRISPR-Cas9. Cas9 mRNA and sgRNA were loaded into exosomes by electroporation resulting in a gene silencing efficiency of approximately 32%, implying that non-nucleated cell-derived exosomes may be useful carriers for delivering mRNA.

Other non-viral vectors reported in the literature for CRISPR-Cas9 delivery are zeolitic imidazole frameworks, silica nanoparticles, and graphene oxide structures [69–71].

Among the non-viral vectors, LNPs are the most widely studied as promising vectors for nucleic acids. The approvals of Onpattro™ and the mRNA-based COVID vaccines have inspired researchers to discover additional safe and effective non-viral vectors for gene therapy [48]. Therefore, LNPs are currently also extensively applied in the CRISPR-Cas9 delivery field. Their advantage is that they can efficiently encapsulate and protect all three forms of the editing system (see 2.3) thus promoting genome editing with different cargos.

## 3 LNPs

LNP sin a broader sense represent a class of lipid-based nanosized systems, including liposomes, lipoplexes, and stable LNPs designed for nucleic acid delivery, previously referred to as stable nucleic acid lipid particles (SNALPs). Liposomes are lipidic structures composed of one or more phospholipid bilayers surrounding an aqueous core. The first formulation of nucleic acid loaded liposomes dates back to the 1970s, reporting low encapsulation efficiency due to the use of neutral lipids and the passive encapsulation method. Lipoplexes result from the electrostatic interaction between cationic lipids and nucleic acids. Widely studied *in vitro,* lipoplexes showed low potency, fast aggregation in blood, immune system activation, and premature dissociation [48].

In the area of nucleic acid formulation and delivery, the term LNPs is mainly used for stable nucleic acid lipid nanoparticles; therefore, this review in its context follows this narrow definition.

**Figure 2:**
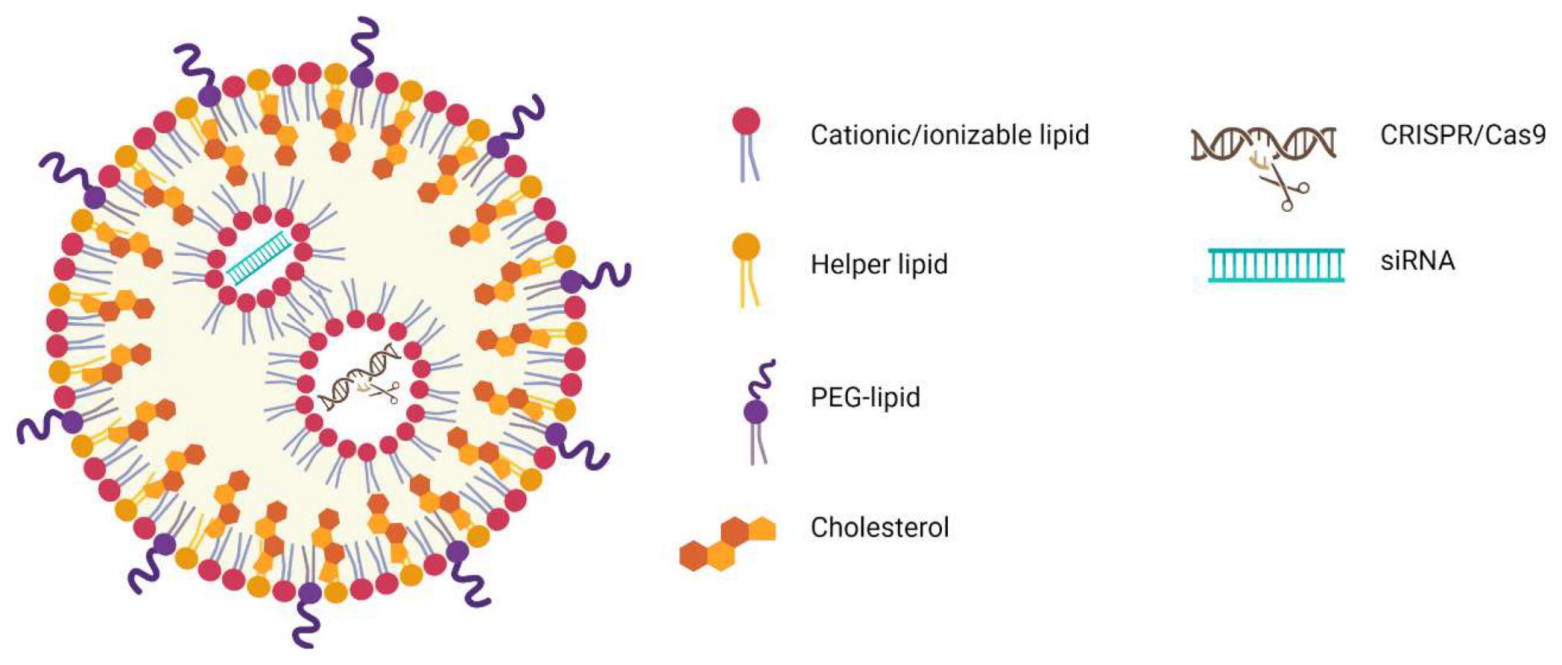
Illustration of LNP formulations for CRISPR-Cas9 and siRNA delivery. The main components of LNP formulations are cationic/ionizable lipids, helper lipids, PEG-lipids, and cholesterol. Cationic/ionizable lipids enable the efficient loading of CRISPR-Cas9 and/or siRNA into LNPs.

In this regards, LNPs are composed of four different lipids:

(i), ionizable cationic lipids, (ii) polyethylene glycol (PEG)-modified lipids, (iii) zwitterionic phospholipids, and (iv) cholesterol [55]. One of their characteristics is their precise and controlled morphology (Figure 2). These systems can efficiently encapsulate both proteins and nucleic acids resulting in one of the most promising non-viral vectors for CRISPR-Cas9 delivery. The first validation of LNP as a clinical drug DS culminated with the approval of Onpattro™ in the US and EU (2018), which is composed of lipid-based structures delivering siRNA for the treatment of hereditary transthyretin-mediated amyloidosis. Even before the success of Onpattro™, LNPs had been investigated for formulation of numerous types of RNA. Several reports of promising data can be found in the literature [72–75]. The previous approval of Onpattro™ also helped the fast development of two LNP-based mRNA-loaded vaccines for intramuscular injection against SARS-CoV-2 spike protein (BNT162b124 and mRNA-127323) for protecting against severe lung infection during the COVID-19 pandemic [53,54,76,77].

### 3.1 LNP components

#### 3.1.1 Ionizable lipids

The success of LNPs is directly associated with the advent of ionizable lipids which have become key elements in ensuring LNPs potency [77]. Ionizable lipids present an amino group (aminolipids) with an acid dissociation constant (pKa) between 6 and 7. The pKa of aminolipids is crucial for the success of the formulation. In particular, in acidic conditions (pH<7) The positively charged amines interact electrostatically with the negatively charged phosphate groups of the nucleic acids (Figure 2). Therefore, LNPs prepared in acidic conditions allow the formation of an electron-dense core that exhibits high payload encapsulation efficiency. By increasing the pH to 7.4, the obtained LNPs show a neutral surface charge thus resulting in a formulation with a low toxic profile and an increased circulation time compared with cationic delivery systems following systemic administration [28]. This mechanism enables to suitably escape the endosomal compartment of the targeted cells. Indeed, endosomes are acid cellular organelles where ionizable lipids present in LNPs become positively charged and electrostatically interact with the strong negative lipids in the endosome membrane thus releasing the nucleic acids into the cytoplasm, where they can carry out their therapeutic actions [28,74,77–79]. Regarding the ionizable lipid design, it was disclosed that by increasing the degree of unsaturation in the lipid chain, the ability of LNPs to merge with endosomes and deliver their payload was further improved. The pKa is adjusted and the composition is modified by customizing the lipid to obtain biodegradable compounds. LNPs composed of ionizable cationic lipids are broadly reported in the literature to enhance the development of innovative gene therapies based on mRNA and CRISPR-Cas9 [80–85].

#### 3.1.2 PEGylated lipids

PEG is a versatile compound which is able to hide the therapeutic from recognition by the immune system and increase its circulation time in the body [58]. PEG-lipids are composed of the hydrophilic PEG domain and the hydrophobic lipid anchor. PEG’s function is manifold and it was traditionally used to prolong the circulation of nanoparticles that were administered intravenously by preventing opsonization. However, PEG also improves the colloidal stability of nanoparticles which notoriously tend to aggregate. When used for LNP formulation, it is necessary to carefully design the carrier aiming to balance the circulation time and the uptake kinetics. Indeed, prolonged PEGylated LNPs circulation may also promote immune system activation. Moreover, PEGylation can interfere with the endosomal escape by impeding LNPs fusion with the endosomal membranes. Therefore, as reported by Semple *et al.* [80], by keeping PEG at a minimum concentration (ranging from 1.5 to 10 mol%) the potency of LNPs improved 5 times [77,86,87].

#### 3.1.3 Phospholipids and Cholesterol

Both phospholipids and cholesterol impact the structure and behavior of LNPs. Known as helped lipids, they contribute to the stability of LNPs, enhancing the transfection activity and facilitating intracellular trafficking. Moreover, they play an important role in the membrane fluidity of lipid-based nanostructures and help maximize the payload encapsulation efficiency [54,77].

### 3.2 LNP preparations and application

In the early 2000s, the advent of the Ethanol Dilution technique revolutionized the LNP field. This technology involves the solubilization of lipids in ethanol which are then mixed, under specific conditions, with an acidic aqueous solution containing the payload. Ionizable lipids suitably interact with nucleic acids, and ethanol is consequently diluted within the aqueous buffer. The encapsulation efficiency of the obtained LNPs was higher if compared with other techniques such as the passive encapsulation of payloads by hydration of a “thin film”, and the LNPs were stabilized only by increasing the pH under physiological conditions [77]. More recently, aspects such as reproducibility and scalability of the Ethanol Dilution method were improved by working at microscale conditions. Indeed, as demonstrated by different authors, the microfluidic mixing approach allowed to synthesize reliable LNPs, maximize their homogeneity and the cargo encapsulation efficiency [88–90].

As many preclinical examples, also Onpattro™ is manufactured by the Ethanol Dilution technique [28,77,79]. The exact composition of this benchmark formulation originates from a study in which fifty-six aminolipids were synthesized and used in combination with distearoylphosphatidylcholine, cholesterol, and (R)-2,3-bis(octadecyloxy)propyl-1 (methoxy poly (ethylene glycol)2000) propylcarbamate to formulate LNPs for siRNA delivery in an LNP library approach. The purpose was the selection of an optimized LNP formulation that, after intravenous administration, efficiently silenced the gene encoding for factor VII. The screening of the lipids resulted in the selection of dilinoleylmethyl-4-dimethylaminobutyrate (DLin-MC3-DMA), with a pKa of 6.2-6.5, one of the most valuable ionizable lipids to efficiently interact, encapsulate and deliver siRNA so far. The use of helper lipids such as cholesterol, 1,2-Distearoyl-sn-glycero-3-phosphocholine, and PEGylated lipid PEG-C14 (molar ratio 1.5% PEG-C14 and 50% DLin-MC3-DMA) efficiently increased the stability of the formulation. PEG-C14 contains short acyl chains, which gradually dissociate from LNPs [91] during blood circulation. The removal of the PEG layer promotes the binding of Apolipoprotein E (ApoE) to the LNPs’ surface which accumulate in the targeted liver tissues. The uptake of LNPs into hepatocytes is mediated from the interaction between ApoE on the nanoparticles surface and ApoE receptors expressed on hepatocytes cellular membrane. When inside the cells, LNPs are protonated due to the acid endosome environment. Consequently, positively charged LNPs and negatively charged endosome membrane lipids electrostatically interact resulting in the disintegration of LNPs, destabilization of the endosome membrane, and the siRNA release into the cytoplasm for therapeutic gene silencing [77,92].

### 3.3 LNP and CRISPR-Cas9

As mentioned above, LNPs are valuable candidates for CRISPR-Cas9 delivery in all three forms. Table 1 summarizes LNP formulations and their applications that are described in the literature for CRISPR-Cas9 delivery in different forms.

These formulations can be modified to improve the carrier’s activity, increase its targetability and promote cellular uptake, thereby overcoming delivery hurdles connected with CRISPR-Cas9 as well as with DNA and RNA delivery in general.

In general, the main disadvantage observed with the delivery of CRISPR-Cas9 as a RNP through LNPs was cargo adsorption on the carrier surface allowing nuclease degradation and immune system recognition after systemic administration [28]. However, new approaches confirmed that LNPs can effectively facilitate the delivery of CRISPR-Cas9 as anRNP, also enhancing its cellular internalization otherwise limited by size and negative surface charge. This was demonstrated by Wang *et al.* [93] who studied and synthesized innovative bioreducible LNPs able to efficiently encapsulate and deliver CRISPR-Cas9 into the cellular nuclei. This approach allowed to improve cargo stability, endo-lysosomal escape and prevented RNP adsorption on the LNPs surface thus, limiting activation of the immune system. However, the presence of nucleic acids which were not part of the RNPs on the surface of the LNPs triggered an immune system response and subsequent RNA degradation [55,94].

During mitosis, delivery of CRISPR-Cas9 in form of DNA encapsulated in LNPs has been evaluated to enter cell nuclei. Kulkarni and colleagues [95] formulated CRISPR-Cas9 DNA into LNPs made of different ionizable lipids and helper lipids. The formulation successfully transfected embryonic mesenchymal cells with a tolerable cytotoxic profile. Further, LNPs for CRISPR-Cas9 DNA delivery were optimized for obtaining improved encapsulation efficiency, cellular uptake, CRISPR-Cas9 expression, and genome editing efficiency [55]. For example, Zhang et al. [96] developed PEG-phospholipid-modified cationic LNPs able to overcome CRISPR-Cas9 DNA limitations such as low encapsulation or cell membrane permeation. Briefly, they used chondroitin sulfate and protamine to form a compact LNP core to increase the plasmid encapsulation efficiency. Moreover, by modifying the ratio between cationic lipid and helper lipid (optimal at 0.8:1), a big impact in terms of LNP’s size, homogeneity, and charge surface was detected. The use of PEGylated lipids improved the stability of LNPs reducing their toxicity and immunogenicity.

In a similar approach, LNPs for the delivery of Cas9-encoding mRNA together with sgRNA targeting antithrombin in the liver were optimized in terms of chemical-physical properties and selectivity due to the heavy modification of sgRNA [28]. The antithrombin inhibition allowed to successfully restore a healthy bleeding phenotype in mice and it was the first approach to treat hemophilia based on CRISPR-Cas9 delivered with non-viral vectors [97]. Other recent reports have demonstrated the feasibility of delivering LNP-encapsulated Cas9 mRNA and sgRNA in mice. Finn and collaborators [98], studied LNPs able to achieve significant and durable *in vivo* CRISPR-Cas9-mediated gene editing, after one systemic administration, relevant for the treatment of liver-based genetic diseases.

Cheng and colleagues [99] designed Selective Organ Targeting (SORT) LNPs for CRISPR-Cas9 mRNA and sgRNA delivery aiming to reach the lungs, spleen, and liver and selectively modify therapeutically relevant cells such as T cells, B cells and hepatocytes. Kenjo and colleagues [54]studied a new therapeutic approach for the treatment of Duchenne muscular dystrophy. They developed pH-dependent ionizable lipids with three hydrophobic tails employed to formulate LNPs for the transient delivery of Cas9 mRNA and sgRNA to the skeletal muscle tissues *in vivo.* This therapeutic approach allowed the repeated injection of LNPs to cover all skeletal muscle. Moreover, investigating the limb perfusion injection method (interesting for the targeting of multiple skeletal muscle groups), LNPs were administered in smaller volumes, compared to conventional hydrodynamic injection, due to the enhanced release of CRISPR-Cas9 from LNPs. Considering the treatment of patients, the injection of small volumes is a huge advantage to avoid compartment syndrome. In comparison to approved antisense oligonucleotide drugs, the effect of LNP-delivered CRISPR-Cas studied by Kenjo was maintained over months. Interestingly, the treated cells did not show mutagenesis overcoming the off-target limitation; however, different candidate DNA cleavage sites were identified.

#### 3.3.1 Application to the lungs

LNPs have been demonstrated to have conducive features to deliver CRISPR-Cas9, in all three forms, to several organs including the lungs. Being an environmentally-connected organ, the lungs are protected by specialized structures and physiological mechanisms such as alveolar macrophage engulfment and mucociliary clearance [107]. Therefore, due to the presence of physiological barriers (see *4.1.),* the delivery of substances to the lungs is challenging [107,108]. So far, the only *in vivo* studies for the treatments of lung pathologies by gene editing were focused on the systemic administration of LNPs delivering CRISPR-Cas9. Examples are investigations carried out by Parhiz and colleagues [109] or the SORT approach which involves the systemic administration of LNPs and exploiting the natural mechanisms of redirecting substances to specific organs. The strategy consists of administering LNPs composed of permanent cationic or anionic lipids and modulating their molar percentage. Cheng and colleagues confirmed that by increasing the amount of a specific cationic lipid in the formulation, LNPs were principally redistributed to the lungs [77,99]. A similar approach was investigated by Wei and Colleagues who obtained LNPs targeting the lungs by adjusting the molecular components and ratios to obtain organ-specific mouse models of cancer and to therapeutically restore gene expression in the muscle [100].

Notwithstanding the interesting results in the aforementioned studies, literature widely reports that systemic administration of drug DSs carrying CRISPR-Cas9 is not the most suitable strategy for lung therapy. Indeed, after systemic administration, several off-target effects and thus toxic conditions have been observed resulting in poor therapeutic outcomes [108].

Therefore, despite the challenging biological barriers to be overcome, the local administration of CRISPR-Cas9 remains the best route for pulmonary treatment. Indeed, inhalation and aerosol have improved the therapeutic efficacy of many cargoes to promote its retention and accumulation in the lungs thus limiting off-target and toxic effects as reviewed by [110] and [111].

An interesting study reporting the successful local delivery of LNPs to the lungs following nasal administration was carried out by Robinson *et al.* who developed LNP delivering CF transmembrane conductance regulator mRNA for the treatment of CF [108]. Despite the delivery approach proving its efficacy for gene editing, there is still a gap between bench research and clinical translation that requires optimization on the delivery and administration side. LNP-CRISPR-Cas9 directly delivered to the lungs by exploiting local administration could therefore be a suitable approach to achieve the best outcomes in terms of lung pathology treatment and/or diagnosis.

## 4 Pulmonary administration

### 4.1 Challenges in the delivery of nucleic acid-based cargoes to the lungs

Intravenous or intramuscular administration of formulations designed for gene therapy to treat lung diseases is associated with several drawbacks, mostly related to the short-term stability of the carriers under physiological conditions. Non-viral vectors loading RNA-based cargoes developed for systemic administration are prone to aggregation with serum proteins, phagocytosis, and rapid renal clearance, which culminates in significantly reduced efficacy [112,113]. Moreover, they tend to accumulate in the liver upon intravenous injection and the dose that effectively reaches and is retained in the lungs is often low. Accumulation in lung capillaries is often a sign of aggregation and capillary blockage and does not lead to productive delivery to the lung parenchyma [114]. The pulmonary route has been investigated as an alternative to overcome these challenges and ensure efficient local delivery [12,115,116]. This route enhances therapeutic efficacy by delivering the nucleic acid-based cargoes directly to their site of action with increased concentration and reduced systemic exposure, diminishing cargo loss and adverse effects. The pulmonary administration benefits from a large surface area (around 142 m^2^) highly vascularized, a thin air-blood and highly permeable membrane (0.2–0.7 μm thickness in the alveolar range), no first-pass metabolism, negligible degrading enzyme activity, and is a non-invasive route [117,118]. All those advantages make it very attractive for both local and systemic administration of gene therapy.

**Figure 3:**
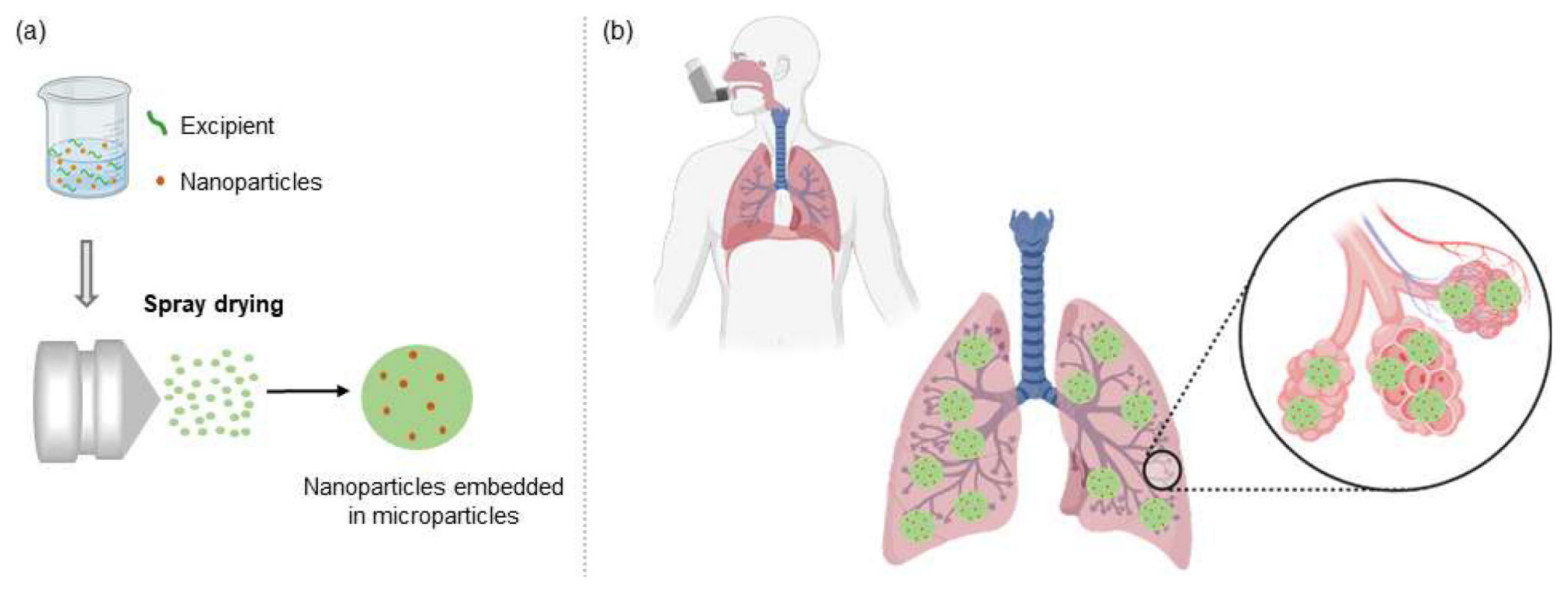
Schematic representation of NEM preparation and pulmonary delivery. (a) Nucleic acid-loaded nanoparticles are mixed with an entrapping matrix excipient to ensure the preparation of dry powders by spray drying. (b) NEMs are loaded into a dry powder inhaler and, due to ideal aerodynamic properties, the formulation can penetrate the lungs and release RNA-based nanoparticles at the target site.

Although the pulmonary administration of RNA-based nanoformulations overcomes part of the challenges imposed by the intravenous route, some consistent and tricky inherent lung barriers still need to be surpassed to ensure safe and efficient delivery. Anatomically, the highly branched structure of the lungs and the tightly-joined epithelium of the airways are natural effective barriers to preventing the penetration of large particles into the lungs [119]. Microparticles larger than 5 μm that are directly administered to the lungs reach high velocity induced by gravity and therefore, are dominated by early inertial impaction that consequently retains them in the oropharyngeal area [120]. On the other hand, nanosized particles are prompt to be exhaled upon inhalation, due to the Brownian motion that maintains them suspended in the respiratory tract and does not allow a proper passage through the lungs. According to the literature, ideal aerodynamic sizes to ensure particle deposition in the deep lung are between 1-5 μm [121]. Therefore, to benefit from the unique and potent properties of RNA-based nanoparticles in the lungs, they must be embedded into microparticles for further pulmonary administration. While in contact with the lung fluid, microparticles may be dissolved upon impaction and thus, release nanoparticles [6]. The idea behind nano-embedded microparticles (NEM) is to deliver nanoparticles to the deep lungs taking advantage of microparticles composed of a matrix carrier with appropriate aerodynamic properties (Figure 3). Indeed, this strategy has been successfully used in the past few years to ensure penetration through the lungs and nanoparticle efficacy [12,122–124].

After overcoming the anatomical challenge, nanoparticles in general, including LNPs, need to pass through the mucus lining to successfully ensure aerosol delivery. The airway mucus is a complex mixture with a gel-type texture that is essentially composed of mucin, water, salts, non-mucin proteins, DNA, and cells, among other constituents. Mucins are glycoproteins that confer a highly negative charge provided by the glycol side chains at physiological pH [119]. Considering that nanoparticles developed for gene therapy are mainly cationic, they tend to being retained within the mucus layer due to electrostatic interactions. Besides hampering the delivery efficiency of nanoparticles, these interactions also affect the stability of nucleic acid-based cargo that will be prone to degradation by the nucleases present in the lung lining fluid. A possible strategy to overcome this issue is to design neutral or negatively charged nanoparticles through hydrophilic PEG coating and adjusting the type and ratio of the lipids used. Indeed, PEG-coated nanoparticles have been reported to exhibit increased mucus penetration [125–127]. On the other hand, we recently demonstrated that, depending on the nanoparticle composition, PEGylation does not always improve mucus penetration [128]. Additionally, PEG coating is likely associated with decreased nanoparticles binding with the negatively charged cell membranes, as discussed under 3.1.2, which consequently culminates in reduced transfection levels and limited cellular uptake [116,129]. This so-called ”PEG Dilemma” phenomenon raises an important concern about balancing physicochemical properties during the design of nanoparticles to optimize pulmonary delivery. Furthermore, it has been reported that highly negatively charged nanoparticles (-50 mV or more) are surprisingly retained in airway mucus, although an opposite behavior was expected due to repulsive forces. A possible explanation for this contradictory behavior is supported by the hydrophobic interactions between the hydrophobic moieties of both nanoparticles and glycoproteins [116] that again, could be avoided following an adequate formulation design with balanced physicochemical properties.

Another lung barrier that must not be neglected is the disease itself. As extensively reviewed before, CF, COPD, lung cancer, and asthma change the lung environment in to pose additional barriers [5,6,112,130]. CF patients, for example, have dense and highly viscous mucus covering obstructive airways with an associated ventilation deficit [131]. This condition affects aerosol delivery, and particles are prone to deposit in the larger upper airways of the lungs due to the increased local turbulence that prevents particles from deposition in the deep lung [132]. As mentioned above, inflammation is a shared condition for many lung diseases, which favors infections and hence, a more complicated scenario to handle emerges. One concern that may arise from an inflamed lung colonized by bacteria is the reduction in the permeability of tight junctions that boosts the resistance to nanoparticle penetration in the airway epithelium [133]. In addition, the pore size of the lung mucus is expressively reduced in the diseased lung, such as in case of asthma, CF, or COPD. According to [134], a healthy lung mucus has an average pore size range of 100 – 500 nm, which is reduced to 100 nm or less in the presence of the above-mentioned conditions.

This scenario challenges the penetration of nanoparticles through the lungs and reinforces the importance of investing into the engineering of NEMs to ensure effective delivery with appropriate aerodynamic properties. The potential of RNA-based nanoparticles against severe lung diseases has been demonstrated. In a recently published study we confirmed the ability of siRNA-loaded lipid-polymer hybrid nanoparticles to penetrate through artificial CF mucus and sputum samples donated from patients, highlighting the potential of the formulation to overcome the challenging and complex barriers in the CF lungs. Using air-liquid interface culture, *in vitro* gene knockdown was successfully achieved, ensuring the capacity of polymer hybrid nanoparticles in delivering siRNA for effective intracellular targeting. However, the PEGylated formulation did not increase mucus penetration [128], confirming the contradictory effects on PEGylation already reported in the literature [135]. Other groups focusing on the aerosol delivery of gene therapy have conducted *in vivo* studies to elucidate the potential of NEMs as an inhaled therapy. Xu and co-workers [124], successfully developed NEMs composed of lipidoid-polymer hybrid nanoparticles loading siRNA against TNF-α embedded into microparticles, which were made of an excipient mixture of leucine, trehalose, and dextran. Following the *in vivo* pulmonary administration of the dry powder inhaler formulation, results demonstrated that it was evenly distributed throughout the lungs. Noteworthy, the formulation was effectively delivered to the deep lungs and slightly retained in the trachea, compared to the deposition of both liquid and reconstituted dry powder formulations. Since mucociliary clearance actively removes particles deposited in the trachea, these particles were unlikely to remain in the lungs. Moreover, this study highlighted the potential of NEMs to overcome lung barriers and be homogeneously distributed throughout the lung’s surface, which leads to a high cargo bioavailability in the target site [124].

### 4.2 Dry powder formulations for pulmonary administration

The advantages of NEM formulations to the lungs offer further benefits than the important ability to overcome lung barriers. Inhalable dry powder formulations remarkably reduce cargo chemical instabilities and microbiological contamination compared to liquid formulations [136,137]. Physical stability is also ensured by inhalable solid dosage forms since the absence of water favors the conditions for transportation and storage, which may positively impact costs. The relevance of these conditions can be exemplified by the recent Comirnaty® vaccine against SARS-CoV-2-infection, which requires extremely low-temperatures for long-term storage and thus, faced numerous limitations during its roll-out in countries lacking infrastructure for the storage [138]. This scenario clearly emphasizes the necessity of engineering LNP for pulmonary administration with improved physical stability, which could potentially be addressed by the manipulation of NEMs. In addition, dry powder formulations are in general preferred by patients due to many factors that increase patient compliance and are mostly related to the devices, such as the ease of handling, the fact that no power source is needed, and their compact sizes [138].

### 4.3 Spray drying as an alternative for efficient preparation of NEMs

Inhalation technology to ensure safe and efficient delivery to the lungs has been widely explored during the past years. And with it, spray drying has emerged as a powerful technique to produce inhalable sophisticated drug DSs with controllable properties. Indeed, by setting the inherent parameters on the spray drying device and the liquid feed, particles prepared through spray drying may be engineered in terms of particle size, density, flowability, morphology, and moisture content that will further impact the formulation distribution and dissolution throughout the airways [118]. Among its several advantages, spray drying is also referred to as a rapid, continuous, cost-effective, reproducible, and scalable method. The principle of spray drying involves four steps and is based on the atomization of a liquid feed into fine droplets, which are dried through a hot drying gas that ensures solvent evaporation and yields a dry powder collected in a cyclone [12].

Although the use of spray drying for inhalable gene therapy assures manageable tools to obtain NEMs with appropriate aerodynamic properties for lung deposition, the technique also needs to ensure the stability of the nucleic acid-based cargo [139]. Therefore, studying and adjusting spray drying parameters is crucial for nucleic acid-loaded NEM development. In theory, the exposition of the recently formed droplets towards mild to high temperatures during solvent evaporation may affect the integrity of the cargo. However, some authors have reported that since the contact time between droplets and the drying gas is short, the impact on the cargo is marginal even in case of thermosensitive compounds [116]. Chang and co-workers [140]also complement this information by pointing out that the real temperature within the spray droplets is lower than the drying gas temperature, due to cooling during evaporation. Chow *et* al. [141] spray dried naked siRNA-containing excipients to obtain a dry powder with appropriate aerodynamic properties, and preserved siRNA integrity. Even though the literature demonstrates that nucleic acids can be exposed to controlled temperature over spray drying and maintain the cargo integrity, the temperature during the process should be prudently optimized to avoid cargo degradation.

In addition to temperature, other spray drying parameters must be fine-tuned for the development of RNA aerosol particles. Shear forces during atomization have an impact on the ultimate properties of aerosolized particles, such as size, shape, and residual moisture content. Consequently, these properties may affect the aerodynamic performance of dry powders and also the RNA stability, especially if there is still significant residual moisture left [142]. Parameters referring to the feed solution and excipients have similar importance to ensure satisfactory aerodynamic properties with a high powder yield. According to the literature, excipients with a low glass transition temperature tend to produce a viscous dry powder, which is prone to attach to the walls of the chamber or the cyclone, reducing the overall yield [12]. Moreover, the gas flow rate and the pump speed were referred to as parameters that directly affect the dry powder yield, particle size, and residual moisture content [143,144]. The feed flow rate and the feed solution concentration are also parameters that proportionally influence the dry powder yield and therefore, are worth being optimized during the development of NEMs [144].

With a solid knowledge of the importance of spray drying parameter optimization, the technique is a potent tool to boost the translation of inhaled RNA from the bench to the clinics to treat lung diseases. As recently reviewed by Munir and co-workers, clinical and preclinical trials of genetic medicines for pulmonary delivery have focused on nebulization and intratracheal administration methods. This means that formulations are aerosolized as a liquid into the lungs, which may induce inflammation due to the prolonged exposure time compared to inhalation methods. Besides, infections may be favored when a liquid formulation is spread throughout the lungs, and intratracheal administration does not ensure a deep lung penetration to the alveolar area, limiting its applications [116]. Thus, preparing loaded RNA-based NEMs for pulmonary administration as an inhalable powder, by taking advantage of spray drying, has the potential to achieve remarkable milestones in pulmonary gene therapy.

### 4.4 Essential aspects of formulations to be spray dried into NEMs

Preparing a stable RNA-based NEM with conducive diffusion within the airways requires careful optimization of spray drying parameters, but also aspects of nanoparticle optimization from the pharmaceutical technology point of view need to be considered. If spray dried without the addition of any excipients, nanoparticles are highly likely to form aggregates due to their large surface area and the stress imposed by adhesion forces that are increased during the dehydration process [145]. Consequently, the redispersibility will not be satisfactory and the underperforming final product may hamper the penetration and cell uptake efficiency in the lungs. This is the main reason for encouraging the use of stabilizing excipients during spray drying. Generally, non-reducing sugars (*e.g*., trehalose), reducing sugars *(*e.g*.,* lactose), sugar-derived polyols (*e.g*., mannitol), or amino acids (*e.g*., leucine) are recommended as formulation excipients for spray drying to control and diminish destabilization of nanoparticles during drying [145,146]. Although the exact mechanism is not yet clear, leucine, for example, has surfactant properties that reduce the surface tension of the aqueous feed solution during atomization. Consequently, particles obtained by the end of the process have smaller sizes, which may improve the chances of being diffused throughout the lungs [146].

Carbohydrate-based excipients are among the most typically used ones for spray drying regardless of the cargo (see Table 2). The stabilizing effect of sugars in particular is possibly associated with the water replacement mechanism, in which hydrogen bonds between the excipient and water are disrupted upon the drying process, generating a dried amorphous matrix entrapping the cargo. If the glass transition temperature of the sugar is reached, either under storage or during the preparation, the matrix risks converting to a crystalline and more stable form. This transition will potentially create a negative impact on the aerodynamic properties of the delivery system. Reaching the glass transition of the sugar is a more important concern when microparticles directly entrap the cargo, since both the solubility and bioavailability tend to decrease in the crystalline form, reducing the overall efficacy of the formulation [147]. Therefore, a critical evaluation of the ideal excipients and their physicochemical properties for a formulation to be prepared by spray drying is indispensable.

Some studies have highlighted the necessity of optimizing excipients in nanoparticle formulations that will further be embedded into microparticles by spray drying. In a previous project, we have spray dried bulk DNA PEI nanoparticles containing different excipients and demonstrated that nanoparticles were only successfully reconstituted after optimizing concentrations of trehalose and mannitol. When the amount of both excipients was suboptimal, redispersed nanoparticles presented larger particle sizes due to aggregation or reduced redispersibility. We, therefore, suggested that the larger the water loss upon drying, the higher the excipient concentration necessary for efficient nanoparticle reconstitution [122]. A subsequent study with siRNA PEI-nanoparticles confirmed the necessity of optimizing the excipient concentrations to ensure redispersion of nanoparticles with preserved size distribution after spray drying [123].

Some excipients, such as mannitol and sorbitol, have been established as mucolytic molecules, with the ability to improve particle penetration through the mucus. The explanation behind this is based on the osmotic or hydrating properties of these molecules, which emulsify the mucus and reduce its viscosity, facilitating the dispersion of the particles within the mucuslayer [148].

### 4.5 Nanoparticles as inhalable NEM

As a proof of concept, many groups have developed inhalable nucleic acid-based NEMs and demonstrated their suitable aerodynamic properties for lung administration, efficient redispersion, and cargo integrity. Table 2 summarizes some of these studies pointing out their main findings.

The main obstacle for clinical translation of RNA nanoformulations is identifying a potent and safe delivery vector. Table 2 emphasizes the possibility to obtain NEMs made of a variety of nanoparticles. One of the concerns that polycation-based nanoparticles face regarding their establishment as efficient RNA delivery platforms is their potential toxicity. Polyplexes composed of PEI have a remarkable delivery capacity, however, the concern about possible PEI accumulation in the body and further toxicity that limits the translation to the clinics is often raised [12,157]. LNPs, on the other hand, also have an efficient delivery potential, are well-tolerated by patients as confirmed by billions of Covid vaccines doses administered. LNPs are composed of biocompatible lipids that are partially endogenous lipids, as in case of cholesterol. However, cationic lipids can present some toxicity but currently can be replaced by ionizable lipids, which are safer [158]. In addition, several other benefits of using nucleic acid-based LNPs as nanoparticle formulation in NEMs such as ensuring efficient permeation within the airways and delivery have been previously highlighted [159]. However, the challenge in preparing RNA-loaded LNPs by spray drying remains due to the low phase transitions temperatures of lipids in contrast to commonly high spray drying temperatures. We were the first to recently publish about successful dry powder preparation constituted of RNA-LNP via spray drying [160]. A patent application was previously disclosed by the company Translate Bio covering this topic and reporting their invention on spray dried LNPs loading mRNA for efficient delivery only after combining LNPs with polymers[161]. Our recent patent application advantageously does not require additional stabilization of LNPs with polymers [162].

Finally, it is worth mentioning that since no spray dried CRISPR-Cas9-LNP was reported in the literature, based on all individual potential benefits from both platforms, this is an attractive alternative to boost the local treatment of pulmonary diseases. Kim et al.[163], published an interesting study investigating the ability of CRISPR-Cas9 in recognizing a specific KRAS mutation in lung cancer to allow for therapeutic gene editing. After intra-tumoral administration using a viral vector platform to deliver CRISPR-Cas9, the lung tumor growth was suppressed *in vivo,* highlighting the outstanding potential of CRISPR-Cas9 for gene editing. Although the efficacy of the platform was demonstrated, the safety remained unclear since the off-target effects were not investigated in detail, but only with a non-selective method. The authors remarked on this shortcoming by reporting that it is not possible to rule out that CRISPR-Cas9 did not elicit mutations in normal cells, which in turn were not detected by limitations on the methodology used [163]. Thus, for future studies, both efficacy and safety of CRISPR-Cas9 in bystander cells must be investigated, and delivering CRISPR-Cas9-loaded LNPs via pulmonary route targeting lung diseases is highly likely to achieve increased efficacy and reduced toxicity.

#### Expert Opinion

Lung diseases are some of the most lethal and disabling conditions occurring worldwide, resulting from genetic and environmental causes. CRISPR-Cas9 has been defined as one of the most revolutionary and innovative technologies that have opened a new therapeutic era for treating diseases cause by genetic mutations. CRISPR-Cas9 shows several strengths, one of which is its versatility in terms of application, therapeutic functions, and delivery forms and strategies broadly classified into physical, viral-vector, and non-viral vector delivery. In general, physical delivery approaches are difficult to be applied *in vivo* and viral vectors can potentially trigger pathological conditions. This leads to developing customizable non-viral DSs capable of addressing pharmacokinetic limitations based on the CRISPR-Cas9 form and administration route. In these regards, LNPs have attracted strong attention due to their ability to efficiently encapsulate and protect all three CRISPR-Cas9 forms enhancing and supporting genome editing.

Interestingly, LNPs were first designed to reach the liver as a target site. Upon intravenous administration, LNP interactions with blood proteins directly determine their ultimate target, therefore the lipid composition makes them prone to accumulation in the liver. The proposed mechanism states that once reaching the circulation, LNPs are opsonized by ApoE, generating a “protein corona” on their surface that will directly influence their distribution. ApoE is recognized by low-density-lipoprotein receptors, which are largely expressed by hepatocytes which hence attract ApoE-coated LNPs[55]. In our opinion, if other target organs for the application of gene-editing LNPs rather than the liver are aimed for, such as the lungs, the formulation must be locally administered to escape as much as possible from the first pass metabolism and the challenges imposed by the systemic route to reach a targeted effect. Indeed, some research groups are addressing the challenge and demonstrated that LNPs designed for gene editing can accumulate in the muscles following repeated intramuscular administration *in vivo* [54]. Moreover, this study successfully confirmed genome editing efficacy by restoring dystrophin expression due to CRISPR-Cas9 action.

Therefore, to ensure an effective and persistent accumulation of LNPs loading CRISPR-Cas9 in the lungs, we support the idea of engineering them as NEMs to be further administered as a dry powder. Taking advantage of spray drying technology, NEMs will achieve enough stability without damaging the gene-editing cargo and will be manufactured with suitable aerodynamic properties to ensure an effective delivery to the airways. Moreover, the local administration of NEMs increases the chances of cargo accumulation at the target site. After a complete release of LNPs in the airways, they have the potential to overcome all barriers in the respiratory tract and deliver CRISPR-Cas9 to their target cells. Although we know that so far, the delivery rates of CRISPR-Cas9 are very low even after local administration, it has been demonstrated that low amounts suffice to ensure an effective and prolonged gene repair in the targeted tissue. Nevertheless, by investing in the engineering of an optimized carrier, delivery rates and safety can be properly controlled to avoid off-target effects and potentially will allow for clinical genome editing.

Finally, the commercial and pharmacoeconomic aspect of developing spray dried LNPs loading CRISPR-Cas9 to treat lung diseases of high importance. In general, the economic burden associated with the treatment of lung diseases is very high in several countrieswithout achieving satisfactory breakthroughs. For example, in one year, a patient with CF costs around €18,000 in Germany and around €50,000 in the UK. Therefore, the application of innovative, targeted, and effective pulmonary therapies is necessary to simultaneously reduce the cost and/or duration of treatment. According to Yang *et al.* [28], the production of LNPs for nucleic acid delivery is simple, fast, reproducible, and low-cost. This was demonstrated by the rapid production of mRNA vaccines against COVID-19 during the 2020 pandemic. In fact, Moderna developed the vaccine in about 42 days, and Phase I clinical trials started only a few weeks later. Furthermore, compared to other gene editing techniques, CRISPR-Cas9 is cheaper, easily scalable, and extremely versatile. Therefore, it is likely that shortly, genome editing therapies could become the most cost-effective therapeutic approach. Spraydrying represents a further economic advantage due to greater stability of the formulation and lower transport and storage costs. From a production point of view, spray drying is synonymous with reproducibility and accuracy capable of ensuring good scalability from the laboratory to industrial-scale processes. Of course, to produce the innovative drugs discussed in this review, suitable and specialized machinery, environments, and personnel are required. Nonetheless, considering the cost/benefit ratio, we firmly believe that LNPs loading CRISPR-Cas9 administered as a dry powder could represent the future of lung disease treatment.

## Figures and Tables

**Table 1:** Summary of studies developing CRISPR-Cas9-loaded LNPs, the main delivery target, and the administration route

CRISPR-Cas9 Form	Target Gene	Target Organ	Administration route	Ref.
Cas9 and sgRNA	EGFP	Brain	Brain injection	[93]
Cas9 and sgRNA	SV40 polyA	Brain	Brain injection	[100]
Cas9 and sgRNA	Pten	Lung	Intravenous injection	[100]
Cas9 and sgRNA	SV40 polyA; p53; Pten; Eml4; Aik; RB1	Lung	Intravenous injection	[100]
Cas9 and sgRNA	Eml4; Aik rearrangement	Lung	Intravenous injection	[100]
Cas9 and sgRNA	DMD	Tibialis anterior muscle	Intravenous injection	[100]
Cas9 and sgRNA	Pcsk9	Liver	Intravenous injection	[100]
Plasmid DNA encoding for Cas9 and sgRNA	EGFP	embryonic mesenchymal cells	*In vitro*	[95]
Plasmid DNA encoding for Cas9 and sgRNA	Plk1	Implanted A375 tumor	Intratumoral injection	[96]
Plasmid DNA encoding for Cas9 and sgRNA	PLK1	Xenograft tumor	Intratumoral injection	[101]
Plasmid DNA encoding for Cas9 and sgRNA	Pcsk9	Liver	Intravenous injection	[102]
Cas9 mRNA and sgRNA	DMD1	Hindlimb muscle	Intravenous injection	[54]
Cas9 mRNA and sgRNA	Pten; Pcsk9; Plk1; PLK1	Multiple Organs	Intravenous injection	[99]
Cas9 mRNA and sgRNA	PCSK9	Liver	Intravenous injection	[103]
Cas9 mRNA and sgRNA	Pah; TTR	Liver	Intravenous injection	[104]
Cas9 mRNA and sgRNA	Ttr; TTR	Liver	Intravenous injection	[98,105]
Cas9 mRNA and sgRNA	Angptl3	Liver	Intravenous injection	[106]

**Table 2:** Examples of successful inhalable nucleic acid-based NEMs

Formulation to be dried	Key excipients	Drying method	Main findings	Ref.
siRNA-dendrimer nanocomplexes	Trehalose, inulin, mannitol	Spray drying (SD)	After SD: successful reconstitution of nanocomplexes, siRNA integrity and function were preserved.	[149]
siRNA-PLGA nanoparticles	Trehalose, lactose and mannitol	SD	SD parameters were optimized and mannitol as excipient achieved the lowest residual moisture. siRNA integrity was preserved after SD, but gene silencing was not reported.	[150]
siRNA-lipid PLGA nanoparticles	Mannitol	SD	SD did not affect the physicochemical characteristics of nanoparticles; both siRNA and gene silencing activity were preserved in dry powder.	[151]
siRNA solid lipid nanoparticles	Mannitol	Thin-film freezedrying, SD, and conventional shelf freezedrying	Thin-film freeze-drying improved physicochemical and aerosol properties compared to SD or conventional shelf freeze-drying.	[152]
Peptide-DNA nanoparticles	Mannitol	SD	Mannitol concentration, inlettemperature and spray rate had a significant effect on DNA recovery, dry powder size, and peptide-DNA-NEM redispersion.	[153]
DNA-PEI polyplexes and DNA-lipopolyplexes	Poly(vinyl alcohol)	SD	NEMs derived from DNA-polyplexes and lipopolyplexes presented appropriate aerodynamic properties, even without the use of stabilizers. Both NEMs increased transfection efficacy compared to the fresh counterparts and ensured successful *in vivo* transgene expression.	[154]
miRNA-lipid– polymer hybrid nanoparticles (LPN)	-	Nebulization	Aerosolized miRNA-LPN had similar physicochemical properties as before nebulization and suitable aerodynamic properties to ensure lung deposition. After nebulization, the miRNA-loaded LPNs retained their ability to inhibit IL-8 secretion.	[155]
siRNA-loaded human serum albumin	Mannitol	SD	siRNA powder had appropriate aerodynamic properties and up to 78% siRNA was preserved upon SD.	[156]
DNA-PEI polyplexes	Mannitol and Trehalose	SD	NEMs derived from DNA-polyplexes and presented appropriate aerodynamic properties, and redispersability was optimized by the excipient content. NEMs increased transfection efficacy compared to the fresh counterparts and ensuredsuccessful in *vitro* transgene expression. Aerodynamic properties were assessed based on DNA content.	[122]
siRNA-PEG-PCL-PEI and Transferrin-PEI polyplexes	Mannitol and Trehalose	SD	SD temperatures and excipient content were optimized to ensure appropriate aerodynamic properties, siRNA integrity and unaffected polyplex gene silencing efficacy after SD and redispersion for both PEG-PCL-PEI and Transferrin-PEI polyplexes *in vitro* and ex *vivo* in primary T cells. Aerodynamic properties were assessed based on siRNA content.	[123]
siRNA-lipid nanoparticles	Lactose	SD	SD temperatures were optimized for siRNA-LNPs for efficient redispersion, appropriate aerodynamic properties and unaffected gene silencing efficacy *in vitro* and ex *vivo* in human lung tissue.	[160]

## Article Highlights

- Chronic respiratory diseases are a major concern in public health worldwide, however, treatment options are limited and mostly related to palliative therapy with several adverse effects.
- Pulmonary disorders have an established genetic target. Thus, gene therapy has remarkable potential to address an effective treatment. CRISPR-Cas9 technology enables genome editing by efficiently and selectively correcting mutations through deletions and/or insertion in the DNA.
- CRISPR-Cas9 delivery is crucial to ensure its safe and effective clinical use. Lipid nanoparticles have emerged as promising carriers for efficiently delivering CRISPR-Cas9, in all three forms, to several organs including the lungs.
- The local administration of lipid nanoparticles loading CRISPR-Cas9 enhances therapeutic efficacy and reduces off-target disadvantages. However, to ensure an efficient pulmonary delivery is necessary to overcome the lung biological barriers.
- Spray drying is a powerful technique to produce inhalable sophisticated drug delivery systems with controllable properties. This technique can support the delivery of lipid nanoparticles-CRISPR-Cas9 to the lungs through the airways by ensuring effective, safe, and prolonged gene repair in the targeted tissue.

## Abbreviations

COPD: Chronic obstructive pulmonary disease
CF: Cystic fibrosis
CFTR: Cystic fibrosis transmembrane conductance regulator
TNF-α: Tumor necrosis factor-alpha
CRISPR-Cas9: Clustered regularly interspaced short palindromic repeats
DSs: Delivery systems
LNP: Lipid nanoparticles
sgRNA: Single guide RNA
REC: Recognition
BH: Bridge Helix
NUC: Nulclease
PI: Protospacer adjacent motif interacting
PAM: Protospacer adjacent motif
DSB: Double-strand break
NHEJ: Non-Homologous End Joining
HDR: Homology Direct Repair
HITI: Homology-independent targeted integration
AAV: Adeno-associated viruses
PEI: Polyethyleneimine
PEG: Polyethylene glycol
DLin-MC3-DMA: Dilinoleylmethyl-4-dimethylaminobutyrate
ApoE: Apolipoprotein E
NEM: Nano-embedded microparticles
SD: Spray drying
SORT: Selective Organ Targeting
